# Insulin autoimmune syndrome in an occidental woman: a case report and literature review

**DOI:** 10.20945/2359-3997000000078

**Published:** 2018-10-01

**Authors:** Mariella Zaiden Rezende Reis, Virgínia Oliveira Fernandes, Eveline Gadelha Pereira Fontenele, Ana Paula Abreu Martins Sales, Renan Magalhães Montenegro, Ana Rosa Pinto Quidute

**Affiliations:** 1 Universidade Federal do Ceará Universidade Federal do Ceará Faculdade de Medicina Hospital Universitário Walter Cantídio Fortaleza CE Brasil Serviço de Endocrinologia e Diabetes, Hospital Universitário Walter Cantídio - Faculdade de Medicina, Universidade Federal do Ceará (UFC), Fortaleza, CE, Brasil; 2 Universidade Federal do Ceará Universidade Federal do Ceará Faculdade de Medicina Departamento de Medicina Fortaleza CE Brasil Núcleo de Pesquisa e Desenvolvimento de Medicamentos (NPDM), Departamento de Medicina, Faculdade de Medicina, Universidade Federal do Ceará (UFC), Fortaleza, CE, Brasil; 3 Universidade de Fortaleza Universidade de Fortaleza Faculdade de Medicina Fortaleza CE Brasil Faculdade de Medicina, Universidade de Fortaleza (Unifor), Fortaleza, CE, Brasil; 4 Universidade Federal do Ceará Universidade Federal do Ceará Faculdade de Medicina Departamento de Fisiologia e Farmacologia Fortaleza CE Brasil Núcleo de Pesquisa e Desenvolvimento de Medicamentos (NPDM), Faculdade de Medicina, Departamento de Fisiologia e Farmacologia, Universidade Federal do Ceará (UFC), Fortaleza, CE, Brasil

## Abstract

Insulin autoimmune syndrome (IAS, Hirata's disease) is a rare hypoglycemic disorder characterized by spontaneous hypoglycemia associated with extremely high circulating insulin levels and positive anti-insulin antibody results. Thus far, most cases have been reported in Asian countries, notably Japan, with few cases reported in western countries. As a possible cause, it is associated with the use of drugs containing sulfhydryl radicals, such as captopril. This report refers to a 63-year-old female Brazilian patient with a history of postprandial hypoglycemia. After extensive investigation and exclusion of other causes, her hyperinsulinemic hypoglycemia was considered to have likely been induced by captopril. Most cases of IAS are self-limiting. However, dietary management, corticosteroids, plasmapheresis, and rituximab have already been used to treat patients with IAS. In our case, after discontinuation of captopril, an initial decrease in insulin autoantibody levels was observed followed by improvement in episodes of hypoglycemia. Although it is a rare disease, IAS should be considered in the differential diagnosis of endogenous hyperinsulinemic hypoglycemia. Patients with suspected IAS must be screened for autoimmunity-related drugs for insulin. Initial clinical suspicion of IAS can avoid unnecessary costs associated with imaging examinations and/or invasive surgical procedures.

## INTRODUCTION

Insulin autoimmune syndrome (IAS), or Hirata's disease, is a rare hypoglycemic disorder first described in 1970 by Hirata and cols. (1). Most cases have been reported in Japanese and Koreans, and few cases in western countries have been described (2,3). It is characterized by spontaneous postprandial hypoglycemia, high levels of immunoreactive insulin, and anti-insulin antibodies. Anti-insulin antibodies triggered by viruses and drugs bind to insulin and proinsulin, resulting in initial hyperglycemia and further stimulation of insulin secretion. Eventually, antibodybinding capacity is exceeded, and unbound free insulin causes hypoglycemia. Dissociation of antibodies also contributes to hypoglycemia (4).

In a Japanese cohort of 197 patients with IAS, 43% were exposed to drugs with sulfhydryl radicals (5), including captopril, methimazole, and tiopronin (4,6). The mechanism by which these radicals lead to the development of IAS is unknown. This paper describes a case of IAS in a South American patient after use of captopril, the most prescribed antihypertensive medicine in our field.

## CASE REPORT

A 63 year old female Brazilian patient complained of recurrent episodes of postprandial hypoglycemia (registered capillary blood glucose of 47 mg/dL), approximately three hours after breakfast, three times a week, with the following symptoms: palpitation, sweating, shakiness and nausea, dizziness, visual blurring, and sleepiness, which improved after sugar intake. These episodes began five years ago with increased frequency in the past three months. Comorbidities were systemic hypertension diagnosed five years ago, obesity and metabolic syndrome, and regular use of captopril 50 mg/day.

There was no personal or family history of diabetes mellitus or other endocrinopathies, such as insulinoma, or previous use of oral hypoglycemic drugs such as sulfonylureas or exogenous insulin. Dietary recall revealed high consumption of foods rich in simple carbohydrates and fats, with low fiber intake. She had a sedentary lifestyle, BMI of 32.2 kg/m^2^, abdominal circumference of 107 cm, and absence of acanthosis nigricans.

Laboratory results are presented in Table 1. Initial blood glucose (8-hour fast), serum insulin, and C-peptide levels were 91 mg/dL (reference range: 65 – 99 mg/dL), 620.9 μIU/mL (reference range: 2.6 – 24.9 μIU/mL), and 3.65 ng/mL (0.9 −7.1 ng/mL), respectively. Our patient was hospitalized for a prolonged fasting test. After 62 hours of fasting, hyperinsulinemic hypoglycemia was observed with high levels of anti-insulin antibodies (294.8 U/mL; non-reactive < 5 U/mL) (Table 1). Endoscopic ultrasonography and abdominal magnetic resonance imaging showed no pancreatic or extra-pancreatic lesions. Anamnesis and detailed clinical investigation ruled out oral hypoglycemic use. Screening for antinuclear factor, serum protein electrophoresis, serology for viral hepatitis, and other autoimmune diseases were negative.

**Table 1:** Clinical and laboratory results during initial and subsequent visits

Parameter	September 25, 2015	November 6, 2015*	September 20, 2016**	Reference Range	Methods
Fasting blood glucose	91	58	97	65-99 mg/dL	Hexokinase
Serum insulin	620.9	246.5	> 300	2.6-24.9 μIU/mL	Electrochemiluminescence
Serum pro-insulin	–	28.2	–	0.5-3.5 pmol/L	ELISA
Serum C-peptide	3.65	2.6	–	0.9-7.1 ng/mL	Chemiluminescence
Hemoglobin A1c	–	–	5.6	4.1-5.7%	HPLC
Serum anti-insulin antibody	–	294.8	100	Non-reactive < 5 IU/mL	ELISA
Serum anti-GAD antibody	–	2.1	–	Non-reactive < 10 IU/mL	ELISA
Total cholesterol	211	–	230	< 200 mg/dL	Enzymatic
LDL	123.2	–	150	< 130 mg/dL	Friedewald Formula
HDL	51	–	47	< 50 mg/dL	Enzymatic Colorimetric
Triglycerides	184	–	164	< 150 mg/dL	GPO - Trinder

Anti-GAD antibody: anti-glutamic acid decarboxylase antibody; LDL: low-density lipoprotein; HDL: high-density lipoprotein; ELISA: enzyme-linked immunosorbent assay.

* Results obtained in the prolonged fasting test (62 hours) after episode of hypoglycemia accompanied by neuroglycopenic symptoms.

** Results obtained after suspension of captopril. It was discontinued in February 2016 with significant improvement in the symptoms of hypoglycemia.

**Figure 1:**
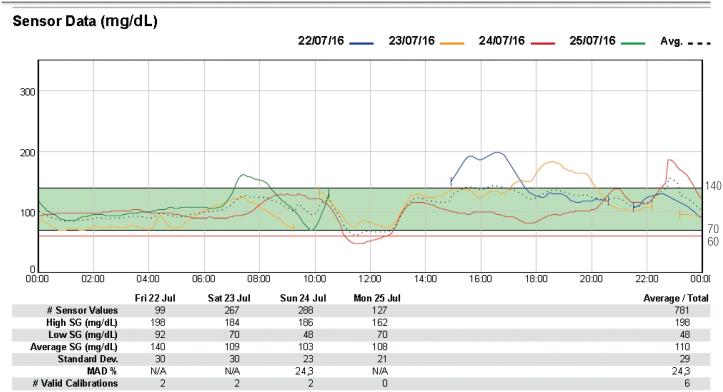
Initial continuous glucose monitoring using the Continuous Glucose Monitoring System: Data of glucose excursions on three consecutive days, 24 h per day. The dotted line represents the average glucose level for three days.

The patient underwent continuous glucose monitoring using the Continuous Glucose Monitoring System (CGMS) (Guardian Real Time^®^; Medtronic, Minneapolis, USA) to detect hypoglycemia within 72 hours. Episodes of hypoglycemia occurred in the late postprandial period (three hours after breakfast) and were concordant with neuroglycopenic symptoms: dizziness, visual blurring, and sleepiness. The lowest glucose value found was 48 mg/dL (Figure 1).

Given high levels of anti-insulin antibodies, hyperinsulinemia, late postprandial hypoglycemia, and clinical presentation coinciding with starting captopril use, the diagnostic hypothesis of IAS was proposed and captopril was discontinued. Six months later, the patient presented a decline in circulating levels of anti-insulin antibodies (100.0 U/mL) and reported reduced frequency of hypoglycemic symptoms (Table 1) to approximately once per month.

**Figure 2:**
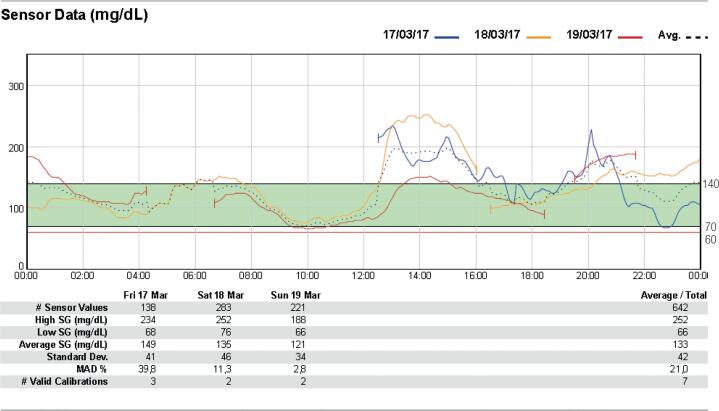
Follow-up continuous glucose monitoring using the Continuous Glucose Monitoring System: Data obtained after 13 months of discontinuation of captopril, for three consecutive days, 24 hours per day. The dotted line represents the average glucose level for three days.

The patient underwent a new CGMS examination (Figure 2), and the lowest glucose value was 66 mg/dL three hours after breakfast without symptoms. The test also revealed sporadic high glucose levels (> 200 mg/dL). A 2-hour 75 gram oral glucose tolerance test (OGTT) was performed; fasting glycemia level was found to be 110 mg/dL, and 2-hour glucose overload was 276 mg/dL. Further workup showed an HbA1c level of 5.6% (Table 1).

Due to this development and presence of metabolic syndrome (7), it was decided to prescribe metformin at the dose of 1000 mg/day, as previously described in another case of IAS (8). At subsequent visits, the patient did not present an increase in episodes of hypoglycemia, nor did worsening of the clinical picture. The patient was also referred for professional nutritional evaluation and dietary plan of 1800 kcal/day, increased fiber intake, reduced lipids, and replacement of simple carbohydrates with complex ones.

In due course, we performed high-resolution HLA class II test and obtained positive results for DRB1*0701, DRB1*0901P, DQB1*0202, and DQB1*0303 haplotypes.

## DISCUSSION

IAS is an uncommon etiology of spontaneous hypoglycemia and was first described by Hirata and cols. in 1970 (1). To our knowledge, this is the fourth case reported in Brazil (8–10). It affects men and women indiscriminately but is more frequent in patients aged > 40 years (11).

Susceptibility to IAS is related to specific HLA class II alleles, which are 10-30 times more prevalent in Japanese and Koreans than in Caucasians (12). Most cases are associated with HLA DR4, DRB1*0406, DQA1*0301, and DQB1*0302 (2,13). Our patient was I positive for DRB1*0701, DRB1*0901P, DQB1*0202, and DQB1*0303. We have not found in the literature other cases with positivity for these haplotypes. In Brazil, Alves and cols. (9) described a case of a seven year old child with HLA DRB1*1104 haplotype, thus far unrelated to the syndrome. Such data suggest that the genetic spectrum of IAS seems to be broader and more heterogeneous than previously thought, leading to the need for further studies to understand the disease in patients from western countries.

IAS may occur with fasting or exacerbated exercise hypoglycemia; however, it is classically characterized by late postprandial hypoglycemia, high insulin levels, and positive results for anti-insulin antibodies.

The pathogenic mechanisms that lead to the development of IAS are not fully understood. They seem to be associated with the formation of insulin- antibody complexes, which hinder the physiological mechanism of insulin action. After a meal, anti-insulin antibodies bind to secreted insulin in response to increased glycemia. This binding reduces the availability of insulin to its receptors in the liver and peripheral tissues causing hyperglycemia and additional secretion of insulin. The hyperglycemic effect is dose-dependent according to anti-insulin levels. Parallel to the decrease in blood glucose, the insulin bound to the antibodies is released, which results in free insulin concentrations disproportionately high for glycemia. Thus, late postprandial hypoglycemia occurs (4,12). Consistent with the kinetics of the insulin-antibody complex in IAS, we could infer that our patient presented with hyperglycemia at the beginning of the postprandial period, explained by the antibodies that bound to the endogenous insulin, with subsequent late occurrence of hypoglycemia when the antibodies dissociated from the insulin, increasing availability. Another possible mechanism behind IAS is the presence of a high- capacity, low-affinity paraprotein, capable of causing hypoglycemia associated with high plasma insulin levels and relatively low C-peptide levels. A plausible mechanism is delayed clearance of insulin, which is still available to bind its receptor due to the relatively weak affinity of the IgA anti-insulin for insulin (4,14).

Initially, we performed magnetic resonance imaging and endoscopic ultrasonography of the pancreas to rule out insulinoma or other extrapancreatic tumors, which would not justify the high levels of anti-insulin antibodies observed in our case. We did not measure plasma sulfonylureas because it was an unlikely diagnosis for our patient. Autoimmune insulin receptor disease (insulin resistance type B) is also part of the differential diagnosis. It is caused by the agonistic effect of antibodies against the insulin receptor resulting in significant insulin resistance and paradoxical hypoglycemia. In these cases, anti-receptor insulin antibodies are usually positive whereas anti-insulin antibodies are negative (4,15). Therefore, considering that our patient did not present acanthosis nigricans, and laboratory results showed high levels of insulin and anti-insulin antibodies, IAS seems to be the most likely diagnosis.

Insulin-immunoglobulin complexes (macrocomplexes) may pose a significant challenge to the measurement of hormones by immunoassay and may also interfere with bioactivity of the hormones to cause clinical disorders. Church and cols. demonstrated that immunoprecipitation with polyethylene glycol (PEG) must be used with caution in screening for insulin-antibody complexes as gel filtration chromatography (GFC) with addition of exogenous insulin enhances sensitivity for the identification of insulin immunocomplexes (16). It is noteworthy that we did not consider the possibility of laboratory analytical interference, and we did not test heterophile antibodies.

IAS is frequently associated with other autoimmune diseases (mainly Graves’ Disease), rheumatologic diseases, previous exposure to exogenous insulin, and use of medications (5,6,12,17). Among drugs, those containing sulfur/sulfhydryl radicals, for example methimazole, captopril, D-penicillamine, hydralazine, glutathione, methionine, mercaptans, imipenem, penicillin G, α-lipoic acid, and diltiazem, have been reported to potentially result in IAS (4,6,18). Our patient did not present any clinical or laboratory evidence of other diseases nor did she have a history of prior insulin use. The only positive finding was captopril use for treatment of hypertension.

Consistent with the pathophysiology of IAS, we have detected late postprandial hypoglycemia in our patient (Figure 1). Once we raised the diagnostic hypothesis of IAS, the medication containing sulfhydryl radical (captopril) was suspended. Six months later, the patient reported improvement in the clinical picture with a decrease in the frequency of hypoglycemic episodes, as shown in Figure 2. In the follow-up CGMS, we also observed hyperglycemia with values > 200 mg/dL throughout the day, which was not seen in the initial CGMS. These findings led us to perform OGTT, and results were consistent with the diagnosis of diabetes mellitus (fasting glucose level: 110 mg/dL; 2-hour glucose level: 276 mg/dL), raising the question of the concomitant possibility of insulin resistance, which could become more evident with the fall in the antiinsulin antibody levels. Our patient had metabolic- syndrome contributing to the clinical picture. Impaired glucose tolerance and overt diabetes do not rule out a diagnosis of IAS, and high HbA1c levels are common in these patients^4^.

Regarding clinical manifestations, reported cases of IAS have varied from mild, transient, to very severe presentations (18). Most patients with drug-induced IAS can achieve remission of the disease soon after stopping use of the medication (6).

Nutritional management is recommended with small, frequent meals and reduction of rapidly- absorbed carbohydrates to avoid rapid elevation of blood glucose levels (18). Alpha-glucosidase inhibitors have also been used to reduce or prevent hypoglycemic episodes (11,12). In more severe or prolonged cases, glucocorticoids, immunosuppressants, and even plasmapheresis may be useful as adjuvant therapy (3,15). Other reported alternatives include pancreatectomy, diazoxide, and octreotide (15). Immunoadsorption using a reusable adsorber system loaded with sheep antigens directed against human immunoglobulin followed by two doses of 1 g of rituximab has been reported effective in a patient with IAS refractory to prednisolone and azathioprine therapy (19). Finally, in the medical literature, meals containing reduced amounts of carbohydrates and corticosteroids were described as the most effective treatments for IAS (11,12).

Because our patient had a mild condition with infrequent hypoglycemia and without severe hemodynamic repercussions, we opted for lifestyle- changes with nutritional dietary management, and suspension of the medication captopril, a known trigger. As previously discussed, to reduce the possibility of concomitant insulin resistance, we initiated the use of metformin. Paiva and cols. reported the use of metformin in the follow-up of a 55 year old Brazilian male patient with suspected IAS with good results (8), which supported use in our patient.

In conclusion, IAS is part of the differential diagnosis of hyperinsulinemic hypoglycemia. It is a rare disease whose pathophysiology is still poorly understood. Initial clinical suspicion of IAS can avoid unnecessary expenses involving imaging examinations and/or invasive surgical procedures, and patients must be screened for autoimmunity-related drugs for insulin. Long-term longitudinal studies are needed to improve understanding of the disease and to improve treatment.
